# Design, Fabrication, and Modeling of a Novel Dual-Axis Control Input PZT Gyroscope

**DOI:** 10.3390/s17112505

**Published:** 2017-10-31

**Authors:** Cheng-Yang Chang, Tsung-Lin Chen

**Affiliations:** Department of Mechanical Engineering, National Chiao Tung University, Hsinchu 30010, Taiwan; bostonchang.me99g@nctu.edu.tw

**Keywords:** cross-axis coupling, dual-axis control input, electrical feedthrough, piezoelectric actuation, piezoelectric gyroscope, piezoelectric position sensing, quadrature error

## Abstract

Conventional gyroscopes are equipped with a single-axis control input, limiting their performance. Although researchers have proposed control algorithms with dual-axis control inputs to improve gyroscope performance, most have verified the control algorithms through numerical simulations because they lacked practical devices with dual-axis control inputs. The aim of this study was to design a piezoelectric gyroscope equipped with a dual-axis control input so that researchers may experimentally verify those control algorithms in future. Designing a piezoelectric gyroscope with a dual-axis control input is more difficult than designing a conventional gyroscope because the control input must be effective over a broad frequency range to compensate for imperfections, and the multiple mode shapes in flexural deformations complicate the relation between flexural deformation and the proof mass position. This study solved these problems by using a lead zirconate titanate (PZT) material, introducing additional electrodes for shielding, developing an optimal electrode pattern, and performing calibrations of undesired couplings. The results indicated that the fabricated device could be operated at 5.5±1 kHz to perform dual-axis actuations and position measurements. The calibration of the fabricated device was completed by system identifications of a new dynamic model including gyroscopic motions, electromechanical coupling, mechanical coupling, electrostatic coupling, and capacitive output impedance. Finally, without the assistance of control algorithms, the “open loop sensitivity” of the fabricated gyroscope was 1.82 μV/deg/s with a nonlinearity of 9.5% full-scale output. This sensitivity is comparable with those of other PZT gyroscopes with single-axis control inputs.

## 1. Introduction

Due to their small size and suitability for batch fabrication [1], vibratory gyroscopes have been extensively used in a variety of applications such as camcorder stabilization, inertial pointing devices, and vehicle antilock brake systems [2]. The conventional operation of a vibratory gyroscope can be briefly described as involving a suspended proof mass moving in a two-dimensional space inside a rigid frame. The frame is attached to an object in motion and rotated with the object. When the proof mass is actuated in a designated direction (called the “Drive” axis) and the object is rotating, as observed from the frame, the Coriolis force would actuate the proof mass in a direction perpendicular to the previous driven direction (called the “Sense” axis). The proof mass motions along both axes are detected to calculate the angular rate of the object [2]. To facilitate such functionality, conventional vibratory gyroscope designs are equipped with a single-axis control input and dual-axis position measurement.

Vibratory gyroscopes are commonly developed using two main approaches: electrostatic and piezoelectric approaches. An electrostatic gyroscope uses electrostatic force and capacitance variation for position actuation and sensing, whereas a piezoelectric gyroscope uses piezoelectricity for position actuation and sensing. Numerous studies have discussed the pros and cons of these two approaches in terms of, for example, interface circuits, process complication, qualify factor, and temperature stability [1,2,3,4,5]. However, few papers have reported that one of the fundamental differences between these two approaches is that the electrostatic approach actuates and senses the position of the proof mass directly, whereas the piezoelectric approach actuates and senses the position of the proof mass through flexure deformation. The flexural deformation and the proof mass position are related by the deformed shape of the flexure. Therefore, the deformed shape of the flexure should contain as few mode shapes as possible to establish a simple relationship between the proof mass position and the flexural deformation. This indirect relationship imposes challenges on and opportunities for designing piezoelectric gyroscopes, which do not exist for electrostatic gyroscopes [6].

**Figure 1:**
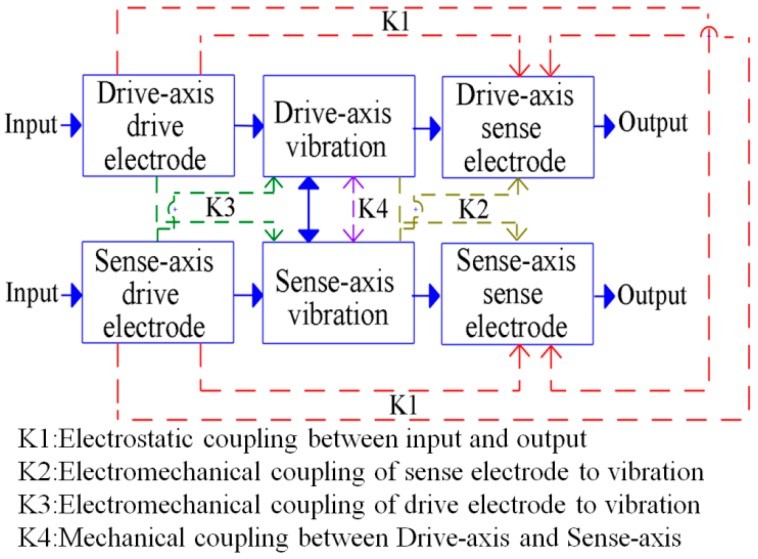
Classification of unnecessary coupling of a dual-axis control input, dual-axis position sensing gyroscope.

In general, three types of coupling would interfere with the gyroscopic motions in piezoelectric gyroscopes: the electrostatic coupling between the input signal and output signal, the cross-axis electromechanical coupling between the electrical signal and proof mass displacement, and the cross-axis mechanical coupling between the proof mass displacements in each axis (Figure 1). The electromechanical coupling mainly comes from the indirect relationship between the proof mass displacement and flexure deformation. Additionally, this coupling can be suppressed by a careful design of the drive and sense electrodes [3,7,8]. The cross-axis mechanical coupling is commonly called the “quadrature error” in vibratory gyroscopes [9] and can be minimized by the orthogonality between two designated resonant motions [7]. The electrostatic coupling is commonly called the “electrical feedthrough”. This coupling is ignored in most gyroscope applications [7,8]. Due to the miniaturized device dimensions and fabrication errors, these unnecessary couplings are two to three orders larger than the gyroscopic coupling; thus, they should be minimized for stable device operation [6,10].

Other than using post-machining to tune the device performance, several papers have proposed using control algorithms to compensate for the effect of those unnecessary couplings [11,12,13]. However, conventional gyroscopes are equipped with only one control input along the Drive axis. Thus, the control algorithm can compensate for the imperfections present on the Drive axis but not on the Sense axis. To improve the performance, researchers have proposed control algorithms that employ dual-axis control inputs to compensate for the errors on both axes. These methods are the force rebalance method [14], dual-axis trajectory following [15,16,17], and direct angle measurement [18]. Because few gyroscopes are equipped with dual-axis control inputs, those previous studies demonstrated an improved performance through numerical simulations but not in their experimental results.

Designing a piezoelectric gyroscope to realize the function of the dual-axis control input can be considerably more difficult than designing a conventional gyroscope with a single-axis control input. This complication occurs because the control input in the dual-axis design must be effective over a broad frequency range to compensate for imperfections, which is not the case for the single-axis design [14]. Moreover, the resonant motion is involved in the structural deformation of the entire device. When one set of drive electrodes is stretching the flexure to generate the proof mass motion, it is likely to interfere with the proof mass motion set by another set of flexures. Additionally, there exist at least two mode shapes with the same amount of amplitude in the proof mass motion in the dual-axis design [3,6,11], which may obstruct the linear relationship between the flexure deformation and the proof mass motion.

The aim of this study was to design a piezoelectric gyroscope equipped with a dual-axis control inputs so that researchers may experimentally verify those control algorithms in future. In our previous design [3], we demonstrated the basics of dual-axis actuation and demonstrated that it is beneficial to sense the proof mass position through flexure deformation. In contrast to our previous work, in this design, we applied separated proof mass, instead of an integrated one, to strengthen the orthogonality between two designated resonant motions. The optimal locations and dimensions of the drive and sense electrodes could be designed accordingly. Additionally, we discuss the trade-off of introducing the ground electrode to suppress the electrical feedthrough. The gyroscope was designed to use a PZT ($\mathrm{Pb}\left({\mathrm{Zr}}_{x}{\mathrm{Ti}}_{1-x}\right)O_{3}$) material, instead of quartz, because PZT materials have a large piezoelectricity and a small quality factor, and because the device fabrication process requires neither sidewall sputtering nor a sandwich structure [4,14]. Finally, we calibrated the fabricated device and developed a dynamic model for the future derivation of control algorithms. The design, fabrication, and calibration of the device are discussed in detail in this paper.

## 2. Conceptual Design of a Dual-Axis Actuation and Position Measurement Piezoelectric Gyroscope

**Figure 2:**
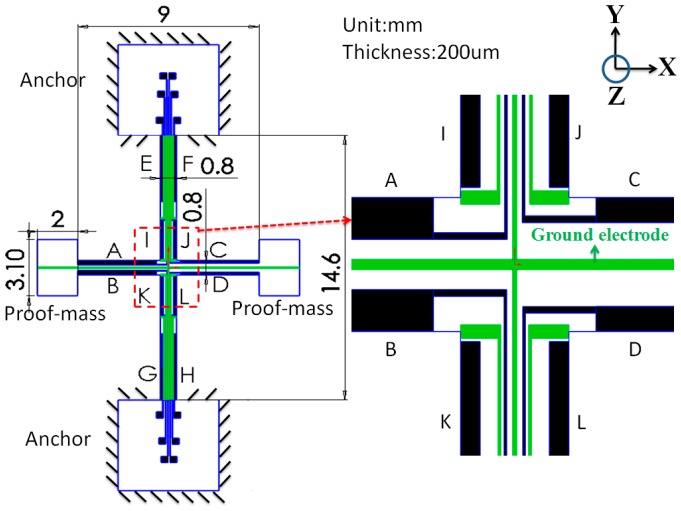
Conceptual design of the proposed gyroscope. The drive electrodes and sense electrodes are drawn in black. The ground electrodes are drawn in green.

Figure 2 illustrates the proposed design, involving a cross-type mechanical structure suspended on the top and bottom. The left and right ends of the structure are the proof mass designated for the gyroscopic motions. The whole structure is made of a bulk piezoelectric material with electrodes patterned on the front and back facets. The same magnitude of voltage is applied to electrodes *A* and *B* (Figure 2), but with different signs, to stretch the flexure and actuate the proof mass in the y-direction. Electrodes *C* and *D* are placed at the right flexure to measure the proof mass motions along the y-direction. Similarly, electrodes *E*, *F*, *G*, and *H* are implemented to actuate the proof mass along the x-direction, whereas electrodes *I*, *J*, *K*, and *L* are used to measure the proof mass motions along the x-direction. The drive electrodes and sense electrodes are separated by ground electrodes to minimize the electrical feedthrough [19]. The electrode on the other side of the structure does not have any pattern and is electrically grounded. The dimensions of the whole device were determined to be 24.6 mm × 13 mm × 0.2 mm including the contact pads. The width of the flexures was determined to be 0.8 mm. 

In the following analysis, a commercial package ANSYS was used to perform the finite element (FEM) simulations to verify the performance of the proposed design including mode shapes, resonant frequency, driving and sensing capability, and angular rate sensitivity. The element used to model the PZT material in these simulations was “SOLID226”, which is 3-D, coupled-filed element capable of modeling the behaviors of, for example, structure-thermal, piezoelectric, electrostatic, and Coriolis force [20]. The convergence error of simulation results was less than 1% with the total number of nodes of 111,817. 

### 2.1. Mode Shape Analysis

**Figure 3:**
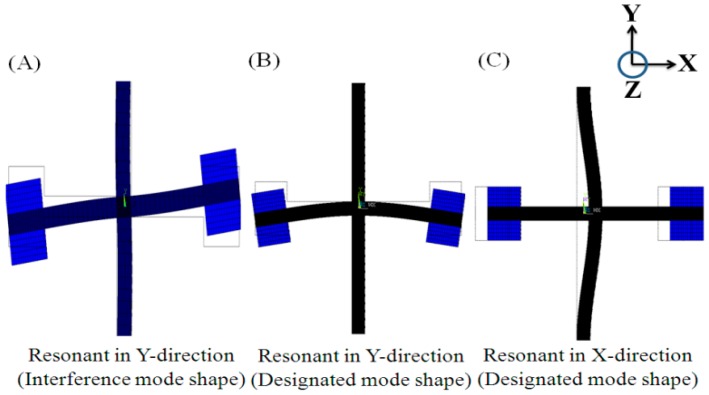
FEM simulation of three resonant motions. Resonant frequencies were 3428, 4893, and 4908 Hz, respectively.

In this study, finite element method (FEM) simulations were used to obtain the resonant modes of the proposed design. As shown in Figure 3 and Table 1, three mode shapes were of interest: (1) the skew-symmetric motion of the proof mass in the y-direction with a resonant frequency of 3428 Hz, (2) symmetric motion of the proof mass in the y-direction with a resonant frequency of 4893 Hz, and (3) lateral motion of the proof mass in the x-direction with a resonant frequency of 4908 Hz. The second and third shapes are the designated mode shapes because the gyroscopic motion would cause energy transfer between these two modes [21]. The existence of the first is likely to result in unnecessary electromechanical coupling and is called the “interference mode” in this paper.

### 2.2. Electrode Design

For the vibration gyroscope to have good sensitivity, both the vibration amplitude of the proof mass and the corresponding displacement-sensing signals should be sufficiently large. As mentioned, vibration actuation and displacement sensing are closely related to the electrode patterns and mode shapes. Because the design involves three types of electrodes and the electrode patterns are different on the front and back facets, the optimal electrode patterns would not exactly match the mode shapes. Therefore, we performed numerical simulations to investigate the optimal electrode patterns.

**Figure 4:**
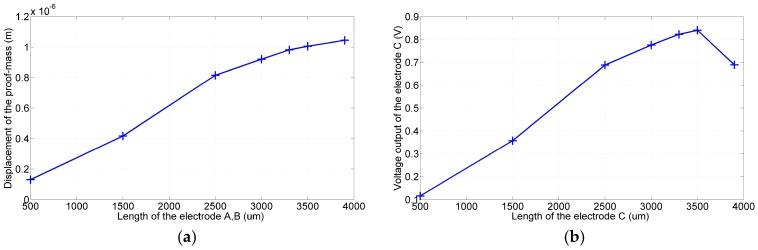
Simulation results: (**a**) Displacement of the proof mass increased with the lengths of the drive electrode pair (*A*, *B*). (**b**) Under the same actuation conditions, voltage output of electrode *C* varied with its length. The optimal length was 3500 μm.

In the first simulation, the backside electrode was grounded and the front side electrodes were given sinusoidal voltages as follows: A=sin(2π×4893t), B=−sin(2π×4893t). The device then vibrated in the designated mode shape, as illustrated in Figure 3 (middle plot). Figure 4a shows that the displacement of the proof mass in the y-direction increased monotonically with the lengths of electrodes *A* and *B*. The larger amplitude implies a more significant gyroscopic motion. Because the total length of the flexure was predetermined by the resonant frequencies of the device, the lengths of electrodes *A* and *B* were both determined to be 3900 μm. 

In the next simulation, electrodes *A* and *B* of length 3900 μm were given the aforementioned voltages. The proof mass then resonated along the y-direction and resulted in a voltage output at electrode *C*. According to the simulation results shown in Figure 4b, this output voltage varied with the length of electrode *C* and reached a maximum value when the electrode length was 3500 μm. The larger output signal implies an improved signal quality for the noise immunization; therefore, the lengths of electrodes *C* and *D* were both determined to be 3500 μm.

**Figure 5:**
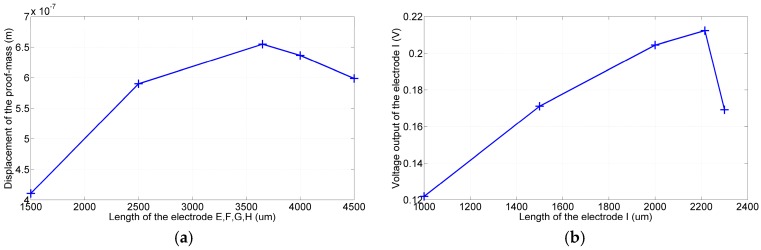
Simulation results: (**a**) Displacement of the proof mass varied with the lengths of electrodes (*E*, *F*, *G*, *H*). The optimal length was 3650 μm for all electrodes. (**b**) Voltage output of electrodes (*I*, *J*, *K*, *L*) varied with their lengths. The optimal lengths was 2215 μm for all electrodes.

Similar approaches were used to determine the lengths of electrodes *E–L.* The electrode pairs (*E*, *F*) and (*G*, *H*) were given the same differential voltages of sin(2π×4908t) to resonate the device in the mode shape shown on the right in Figure 3. The electrode pairs (*I*, *J*) and (*K*, *L*) were used to detect the corresponding proof mass motion. According to the simulation results shown in Figure 5a,b, the optimal lengths of electrodes *E*, *F*, *G*, and *H* and electrodes *I*, *J*, *K*, and *L* were determined to be 3650 and 2215 μm, respectively.

**Figure 6:**
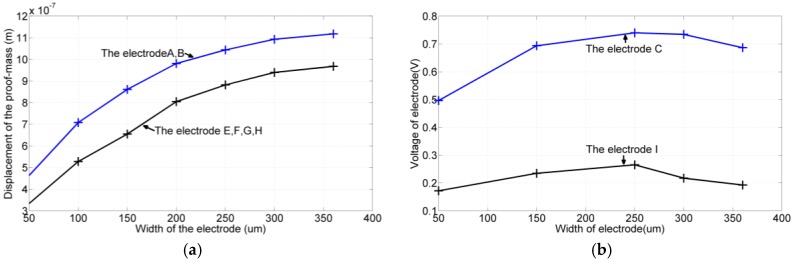
Simulation results. (**a**) Displacement of the proof mass varied with the widths of the drive electrode pairs (*A*, *B*), (*E*, *F*), and (*G*, *H*). (**b**) Voltage output of electrodes *C*, *D*, and *I*–*L* varied with their widths.

After establishing the lengths of the electrodes, we determined their widths. According to the simulation results shown in Figure 6a, the displacement of the proof mass in the y-direction increased monotonically with the widths of the electrode pair (*A*, *B*). Moreover, at the same vibration amplitude, the output voltage of electrode *C* reached the maximum value when the width was 250 μm. Similar approaches were employed to determine the widths of electrodes *E–L*, and similar results were obtained. Although the simulations may indicate the optimal width of each electrode, considering the fabrication capability and reserving an area for the ground electrodes, we determined the widths of the electrode pair (*A*, *B*) to be 250 μm and the widths of electrodes *C*–*L* to be 150 μm. 

Piezoelectric materials are prone to capacitive interference [22]; moreover, in the proposed design, the drive electrodes and sense electrodes are in proximity. Consequently, the electrical feedthrough is a serious matter and could degenerate the angular rate measurement of the device. One possible solution is to shield the sense electrode by using the ground electrode, a common practice in circuit design [23,24]. However, PZT materials exhibit different piezoelectricity levels in different directions. Furthermore, introducing additional voltage potentials to piezoelectric materials is likely to interfere with the designated piezoelectric actuation and sensing. Because the proposed design makes it a bit complicated to elucidate the pros and cons, we could analyze this effect by using a simple cantilever structure (500 μm × 100 μm × 200 μm) instead. 

**Figure 7:**
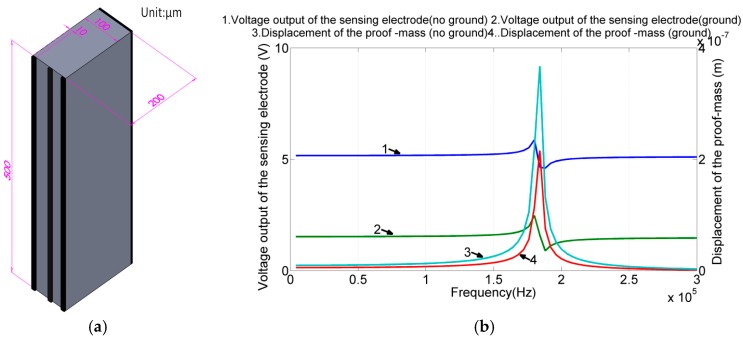
(**a**) Piezoelectric cantilever beam for testing the design of the ground electrode. (**b**) When voltage was applied to the driving electrode, the displacement in the case with the ground electrode was slightly greater than that in the case without the ground electrode. By contrast, the signal at the sense electrode was smaller than that in the case without the ground electrode.

As shown in Figure 7a, three strips of electrodes were patterned on the front: the drive, ground, and sense electrodes. The gaps between the drive–ground and ground–sense electrodes were 40 and 30 μm, respectively. The thickness (200 μm) was the same as the proposed gyroscope design, to produce the same effect from the ground electrode on the back. Figure 7b shows the simulation results of piezoelectric actuation and sensing with and without the ground electrode between the drive and sense electrodes. 

According to the simulation results, the bending motion of the structure with the ground electrode was slightly larger than that of the structure without the ground electrode. By contrast, the signal at the sense electrode was smaller than that in the case without the ground electrode. Assuming the signal at the sense electrode can be attributed to the structure’s bending and electrical feedthrough, we applied curve-fitting techniques to calibrate these two effects. In the case without the ground electrode, the sensitivity of the piezoelectric displacement sensing process was identified to be 6.64 V/μm and the electrical feedthrough was 5.13 V, whereas in the case with the ground electrode, the sensitivity and electrical feedthrough were 4.6 V/μm and 1.49 V, respectively. The decreased sensitivity and enlarged displacement can be attributed to the “d15” and “d32” effects of the PZT material [25]. In summary, with the ground electrode design, we may reduce the feedthrough by 70% at the expense of losing 30% of the sensitivity. 

## 3. Simulations of Device Performance

### 3.1. Driving and Sensing Capability in X-Axis

**Figure 8:**
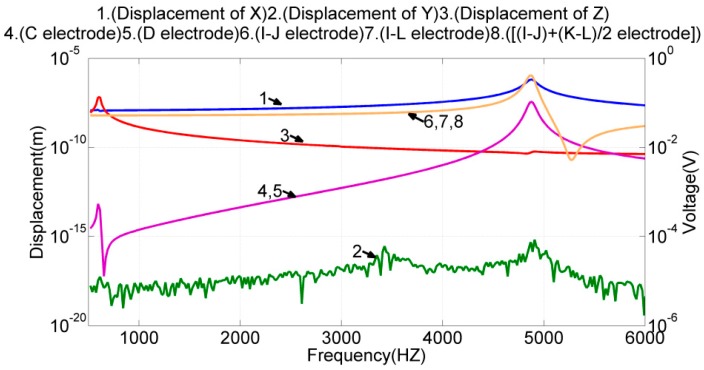
Frequency response of both the proof mass displacement and the voltage output of sense electrodes when the device was actuated by drive electrodes (*E*, *F*, *G*, *H*).

In this simulation, the electrodes (*E*, *F*) were given a sinusoidal voltage with a 1-V amplitude but a 108° phase difference. Electrodes (*G*, *H*) were given the same voltage as electrodes (*E*, *F*). The frequency response of the design was simulated. The results are illustrated in Figure 8; in the illustrated plots, lines 1–3 denote the frequency response of the proof mass displacement along the three axes; lines 4–8 denote the differential voltage outputs of the electrode pairs (*I*, *J*), (*I*, *L*), and (((*I* − *J*) + (*K* − *L*))/2), as well as the voltage output of the single electrode *C*, *D*. Notably, the displacement of the proof mass was taken at the center of the mass block on the right. 

The simulation results indicated that the design of drive electrodes (*E*, *F*, *G*, *H*) could excite only the designated resonant mode in the x-direction, but induced negligible motions in the y- and z-directions. The frequency responses of the three voltage outputs, (*I*, *J*), (*I*, *L*), and (((*I* − *J*) + (*K* − *L*))/2), were almost identical and resembled the frequency response of the proof mass displacement in the x-axis. Therefore, either one is qualified for sensing the proof mass displacement in the x-axis under this actuation. The frequency responses of the voltage output at electrodes *C* and *D* were almost identical. Therefore, their differential voltage output is qualified for sensing the proof mass displacement in the y-axis under this actuation. Moreover, at the resonant frequency of 4.87 kHz, the displacement of the proof mass was 0.4 fm in the y-axis, 0.655 μm in the x-axis, and 0.05 nm in the z-axis. The cross-axis mechanical coupling (=0.4 fm/0.655 μm) was negligible. 

### 3.2. Driving and Sensing Capability in Y-Axis

**Figure 9:**
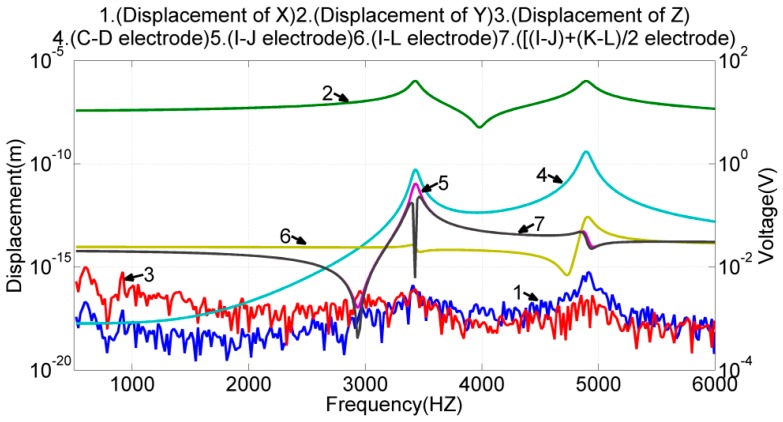
Frequency response of both the proof mass displacement and the voltage output of the sense electrodes when the device was actuated by the drive electrode pair (*A*, *B*).

Similar approaches were used to verify the device driving and sensing capability in the y-axis. In this case, the electrode pair (*A*, *B*) was given a sinusoidal voltage with a 1-V amplitude but a 180° phase difference. The frequency response of the device was simulated, and the results are presented in Figure 9. The simulation results revealed that the design of driving electrodes (*A*, *B*) could excite the interference mode at the frequency of 3.42 kHz, the designated mode at the frequency of 4.89 kHz, and negligible motions in the x- and z-directions. The differential voltage of the electrode pair (*C*, *D*) responded to both resonant modes in the y-direction. Because the frequency response of this pair still resembled the frequency response of the proof mass displacement in the y-axis near the designated frequency, the differential voltage of the electrode pair (*C*, *D*) is qualified for sensing the proof mass displacement in the y-axis under this actuation. The differential voltage of the electrode pair (*I*, *L*) did not respond to the interference mode but did respond to the designated resonant motion in the y-axis. Therefore, the electrode pair (*I*, *L*) is unqualified as a position sensor in this case. The voltage outputs of (((*I* − *J*) + (*K* − *L*))/2) and (*I*, *J*) were insensitive to the designated resonance in the y-direction but did respond to the interference mode. 

According to the analysis of the mode shapes, the voltage output of (((*I* − *J*) + (*K* − *L*))/2) should not respond to the interference mode. The FEM results confirmed this argument but also indicated that the advantage was not obvious. Therefore, the electrode pair (*I*, *J*) was chosen for sensing the proof mass displacement in the x-axis. Moreover, at the designated resonant frequency, the displacement of the proof mass was 1.044 μm in the y-axis, 0.191 fm in the x-axis, and 0.02 fm in the z-axis. The cross-axis mechanical coupling was negligible (=0.191 fm/1.044 μm).

### 3.3. X-Axis and Y-Axis Driving Simultaneously

**Figure 10:**
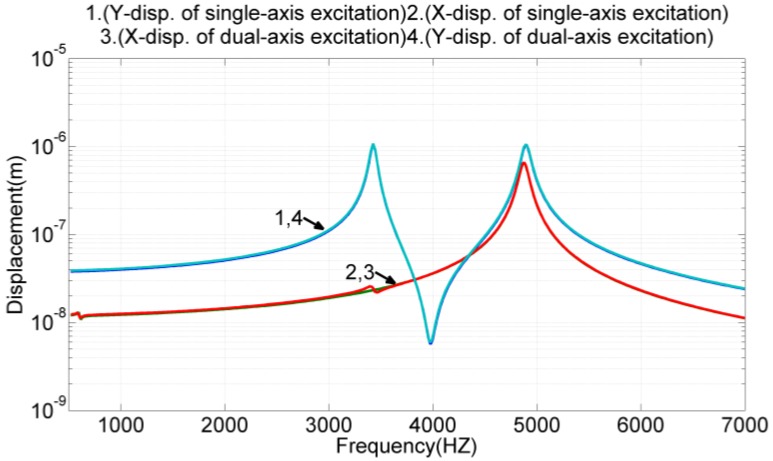
The proof mass displacement under x-axis and y-axis actuations, simultaneously or individually.

In this simulation, the drive electrodes discussed in previous two sections were given the same voltages to resonant the device in x-axis and y-axis, simultaneously. The frequency response of the proof mass displacement was simulated, and the results, along with the results from Figure 8 and Figure 9, were presented in Figure 10 for comparison. The simulation results revealed that the proof mass displacement was almost identical when actuated by the x-axis and y-axis drive electrodes, simultaneously or individually. The difference of two types of actuations was less than 3% in terms of the proof mass displacement.

### 3.4. Simulation of Angular Rate Measurements

**Figure 11:**
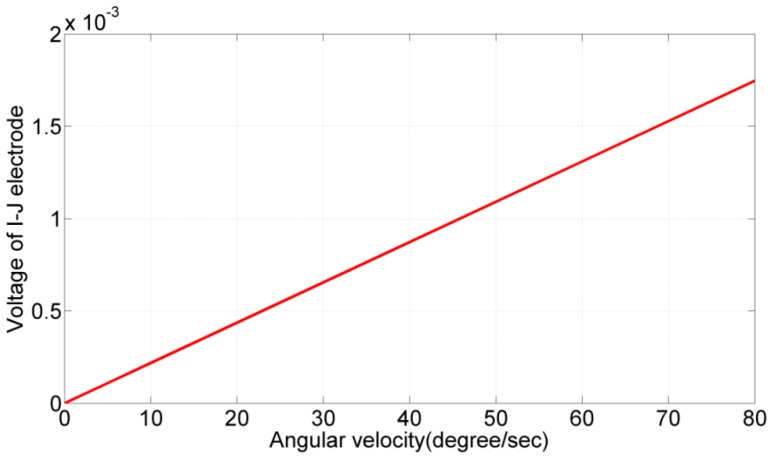
Angular rate measurement and predicted output voltage. The sensitivity was 13.8 μV/deg/s, and the nonlinearity is 0.6% in FSO.

In this simulation, electrodes *A* and *B* were given a sinusoidal voltage with a 1-V amplitude and a 4.89-kHz frequency but a 180° phase difference. The angular rate ranged from 0°/s to 80°/s. The amplitude of the output signal from the electrode pair (*I*, *J*) was recorded, as shown in Figure 11. According to the simulation results, the output signal was linear with the angular rate. The sensitivity was calculated to be 13.8 μV/deg/s, and the nonlinearity was 0.6% in full-scale output (FSO). 

## 4. Device Fabrication

**Figure 12:**
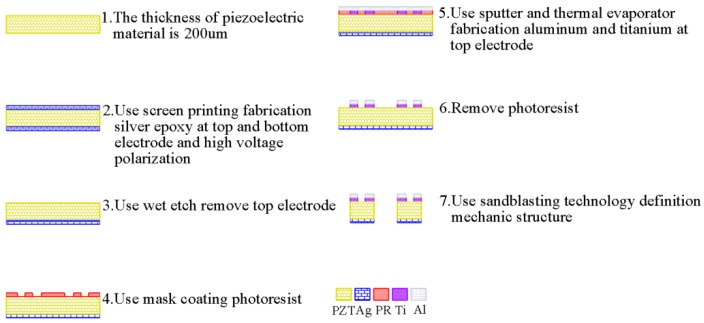
Microfabrication process of the proposed PZT gyroscope design.

**Figure 13:**
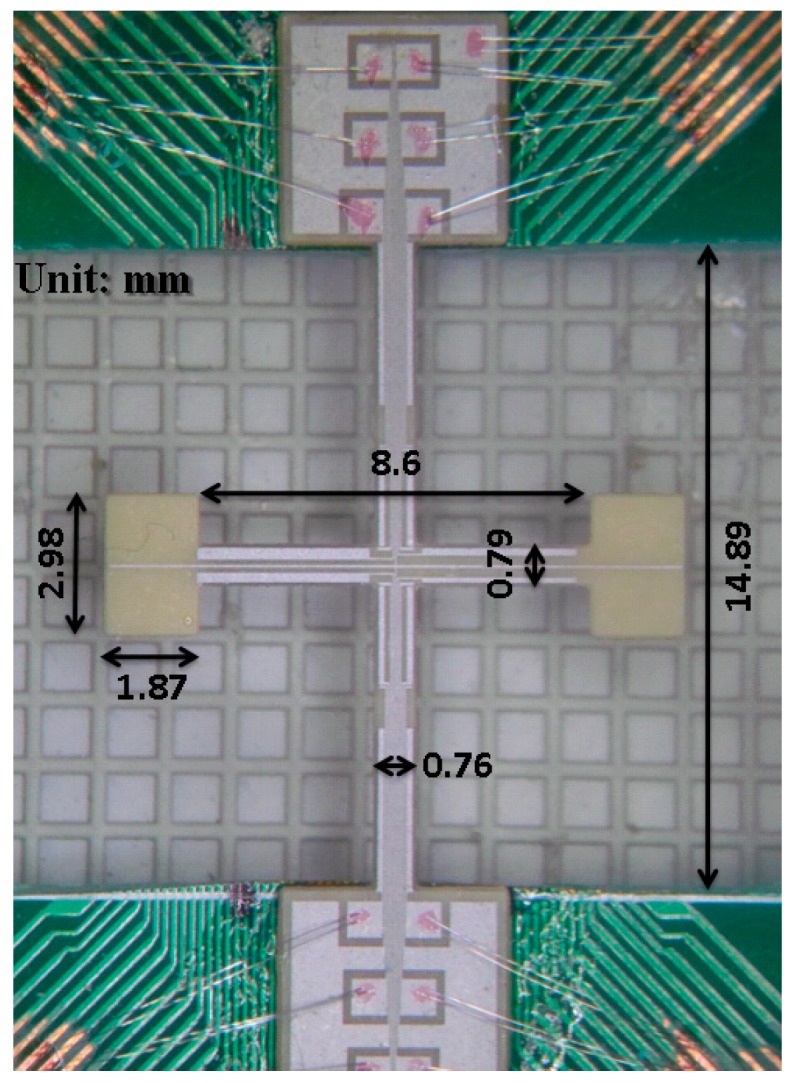
Photo of the fabricated PZT gyroscope.

The fabrication process of the proposed design is presented in Figure 12. The starting material was a bulk PZT-5 (${\mathrm{PZT}\;\mathrm{doped}\;\mathrm{with}\;\mathrm{Nb}}^{5+}\;{\mathrm{or}\;\mathrm{Ta}}^{5+}$) with a thickness of 200 μm. First, a 12-μm-thick silver epoxy was glued on the top and bottom surfaces of the PZT block for piezoelectric polarization. A mixed solution of hydrogen peroxide and ammonia (1:1) was then used to remove the silver epoxy on the top. Second, a 1-μm-thick aluminum was deposited on the top surface using titanium (Ti) as an adhesion layer. The aluminum was then subjected to a lift-off process for electrode patterning. Finally, the mechanical structure of the device was patterned using sandblasting techniques. After the microfabrication process, the fabricated device was glued to a printed circuit board (PCB), and its input and output electrodes were wire-bonded to the pads on the PCB board. A photo of the fabricated PZT gyroscope is presented in Figure 13.

## 5. Experimental Results

In the following experiments, the electrode output voltages were measured by a lock-in amplifier (7265, from Signal Recovery©, Bervin, PA, USA) exhibiting a measurement accuracy of 10 nV. The displacements of the proof mass were measured by a vibrometer (MSA-500 from Polytec©, Karlsruhe, Germany) exhibiting a measurement accuracy of 5 nm.

### 5.1. Dynamic Measurement

**Figure 14:**
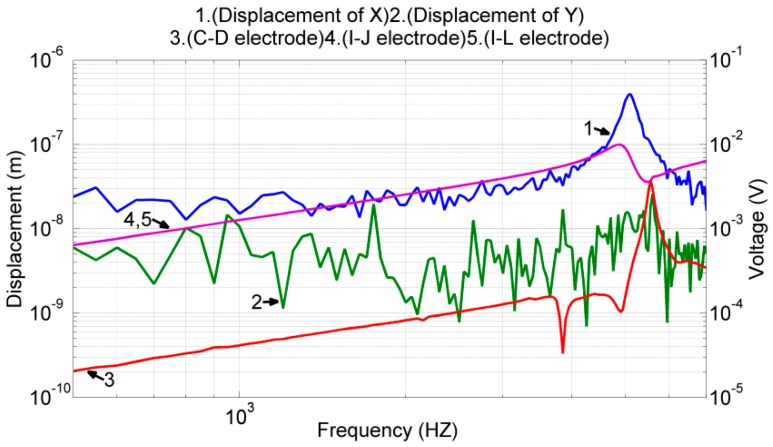
Frequency response of the proof mass displacement and frequency response of the voltage output of the sense electrodes when the device was actuated by drive electrodes (*E*, *F*, *G*, *H*).

In the first experiment, we calibrated the device driving and sensing capability in the x-axis. The test conditions were similar to those described in Section 3.1, except that the voltage output of (((*I* − *J*) + (*K* − *L*))/2) was not measured due to equipment incapability. The experimental results are shown in Figure 14, where lines 1 and 2 denote the proof mass displacement in the x- and y-directions, respectively, and lines 3–5 denote the differential voltage outputs of the electrode pairs (*C*, *D*), (*I*, *J*), and (*I*, *L*), respectively. 

According to the experimental results, the resonant frequency in the x-direction was 5.1 kHz. In addition, near this frequency, the displacement of the proof mass was 13.57 nm in the y-axis and 0.39 μm in the x-axis. The cross-axis mechanical coupling was approximately 3.4% (=13.57 nm/0.39 μm). The frequency responses of the two differential voltage outputs (*I*, *J*) and (*I*, *L*) were determined to be similar to the FEM results shown in Figure 8. The voltage output of the (*C*, *D*) pair and proof mass displacement in the y-axis were slightly larger than those expected near the frequency of 5.5 kHz, which may be caused by the cross-axis mechanical coupling. Additionally, all the voltage outputs exhibit the property of high-pass filtering with a slope of 20 dB/decade. This, and this can be attributed to the capacitive output impedance of the piezoelectric material.

**Figure 15:**
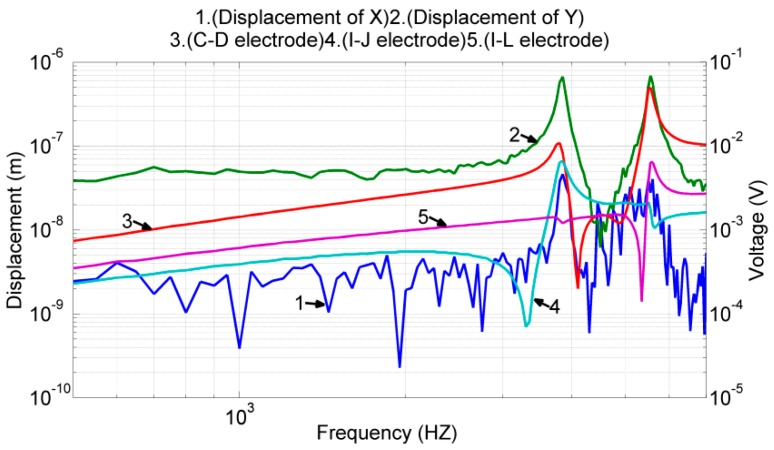
Frequency response of the proof mass displacement and frequency response of the voltage output of the sense electrodes when the device was actuated by the drive electrodes (*A*, *B*).

In the next experiment, we calibrated the device driving and sensing capability in the y-axis. The test conditions were the same as those described in Section 3.2. According to the experimental results shown in Figure 15, the resonant frequency of the interference mode was 3850 Hz, and the designated resonant mode was 5550 Hz. Near the designated resonant frequency, the displacement of the proof mass was 0.68 μm in the y-axis and 39.74 nm in the x-axis. The cross-axis mechanical coupling was approximately 5.7% (=39.74 nm/0.68 μm). The frequency responses of the electrode pairs (*C*, *D*), (*I*, *J*), and (*I*, *L*) were determined to be similar to the FEM results shown in Figure 9. Furthermore, all the voltage outputs exhibited the property of high-pass filtering with a slope of 20 dB/decade.

### 5.2. Calibrations of Dynamics

For the future derivation of control algorithms, we calibrated the device performance to obtain a dynamic model. The fabricated device includes piezoelectric actuation, piezoelectric position sensing, electrical feedthrough, electromechanical couplings, and capacitive output impedance. Therefore, we proposed a model as follows:

(1) $$\left[\begin{array}{c} \dot{x}\\ \ddot{x}\\ \dot{y}\\ \ddot{y}\\ {\dot{v}}_{\mathrm{ox}1}\\ {\dot{v}}_{\mathrm{oy}1} \end{array}\right]=[\begin{array}{cccccc} 0 & 1 & 0 & 0 & 0 & 0\\ \frac{-k_{\mathrm{xx}}}{m_{x}} & \frac{-d_{\mathrm{xx}}}{m_{x}} & \frac{-k_{\mathrm{xy}}}{m_{x}} & 2\Omega_{z}-\frac{d_{\mathrm{xy}}}{m_{x}} & 0 & 0\\ 0 & 0 & 0 & 1 & 0 & 0\\ \frac{-k_{\mathrm{yx}}}{m_{y}} & -2\Omega_{z}-\frac{d_{\mathrm{yx}}}{m_{y}} & \frac{-k_{\mathrm{yy}}}{m_{y}} & \frac{-d_{\mathrm{yy}}}{m_{y}} & 0 & 0\\ -\omega_{H}C_{\mathrm{mx}} & 0 & 0 & 0 & -\omega_{H} & 0\\ 0 & 0 & -\omega_{H}C_{\mathrm{my}} & 0 & 0 & -\omega_{H} \end{array}][\begin{array}{c} x\\ \dot{x}\\ y\\ \dot{y}\\ v_{\mathrm{ox}1}\\ v_{\mathrm{oy}1} \end{array}]+\left[\begin{array}{cc} 0 & 0\\ \frac{C_{\mathrm{vx}}}{m_{x}} & 0\\ 0 & 0\\ 0 & \frac{C_{\mathrm{vy}}}{m_{y}}\\ -\omega_{H}f_{\mathrm{xx}} & -\omega_{H}f_{\mathrm{xy}}\\ -\omega_{H}f_{\mathrm{yx}} & -\omega_{H}f_{\mathrm{yy}} \end{array}\right]\left[\begin{array}{c} V_{\mathrm{inx}}\\ V_{\mathrm{iny}} \end{array}\right]\;\left[\begin{array}{c} V_{\mathrm{ox}}\\ V_{\mathrm{oy}} \end{array}\right]=\left[\begin{array}{cccccc} C_{\mathrm{mx}} & 0 & 0 & 0 & 1 & 0\\ 0 & 0 & C_{\mathrm{my}} & 0 & 0 & 1 \end{array}\right]\left[\begin{array}{c} x\\ \dot{x}\\ y\\ \dot{y}\\ v_{\mathrm{ox}1}\\ v_{\mathrm{oy}1} \end{array}\right]+\left[\begin{array}{cc} f_{\mathrm{xx}} & f_{\mathrm{xy}}\\ f_{\mathrm{yx}} & f_{\mathrm{yy}} \end{array}\right]\left[\begin{array}{c} V_{\mathrm{inx}}\\ V_{\mathrm{iny}} \end{array}\right]$$

where $m_{x}$ and $m_{y}$ represent the equivalent proof mass along the x- and y-axes, respectively; ${\left[\begin{array}{cccccc} x & \dot{x} & y & \dot{y} & v_{\mathrm{ox}1} & v_{\mathrm{oy}1} \end{array}\right]}^{T}$ represent the state of the proof mass position, velocity, and high-pass filtered signal along the x- and y-axes, respectively; $d_{\mathrm{xx}}{\;\mathrm{and}\;d}_{\mathrm{yy}}$ represent the damping coefficients along the x- and y-axes respectively; $k_{\mathrm{xx}}{\;\mathrm{and}\;k}_{\mathrm{yy}}$ represent the spring constants along the x- and y-axes, respectively; $d_{\mathrm{xy}}{\;\mathrm{and}\;d}_{\mathrm{yx}}$ represent the cross-axis damping coefficients; $k_{\mathrm{xy}}{\;\mathrm{and}\;k}_{\mathrm{yx}}$ represent the cross-axis spring constants; $\Omega_{z}$ represents the angular rate along the z-axis; $\omega_{H}$ represent the corner frequency of the high-pass filtering; $f_{\mathrm{xx}}\;-{\;f}_{\mathrm{yy}}$ represent the electrical feedthroughs from the drive electrodes to the sense electrodes; $C_{\mathrm{vx}}{\;\mathrm{and}\;C}_{\mathrm{vy}}$ represent the force conversion factors that convert input voltages into actuation forces along the x- and y-axes, respectively; $C_{\mathrm{mx}}{\;\mathrm{and}\;C}_{\mathrm{my}}$ represent the displacement conversion factors that convert proof mass displacements into output voltages; $V_{\mathrm{inx}}{\;\mathrm{and}\;V}_{\mathrm{iny}}$ represent the differential input voltages applied to the electrode pairs (*E*, *F*, *G*, *H*) and (*A*, *B*), respectively; and $V_{\mathrm{ox}}{\;\mathrm{and}\;V}_{\mathrm{oy}}$ represent the differential output voltages measured at the electrode pairs (*I*, *J*) and (*C*, *D*), respectively. 

The proposed dynamic model comprised 19 parameters, and it may encounter numerical problems when performing conventional system identifications [19]. With the assistance provided by the additional measurement of the proof mass displacement, we proposed a two-stage system identification for this gyroscope system to avoid the numerical problem. In this approach, Equation (1) is converted into the form of transfer matrices to provide submodels compatible with the experimental results. Additionally, similar to most approaches, the cross-axis damping coefficients $d_{\mathrm{xy}}{\;\mathrm{and}\;d}_{\mathrm{yx}}$ are insignificant and could be ignored for simplicity [26,27].

(2) $$\left[\begin{array}{c} V_{\mathrm{ox}}\left(s\right)\\ V_{\mathrm{oy}}\left(s\right) \end{array}\right]=G_{\mathrm{HP}}\left(s\right)\left(\left[\begin{array}{cc} C_{\mathrm{mx}} & 0\\ 0 & C_{\mathrm{my}} \end{array}\right]\left[\begin{array}{cc} G_{\mathrm{xx}}\left(s\right) & G_{\mathrm{xy}}\left(s\right)\\ G_{\mathrm{yx}}\left(s\right) & G_{\mathrm{yy}}\left(s\right) \end{array}\right]+\left[\begin{array}{cc} f_{\mathrm{xx}} & f_{\mathrm{xy}}\\ f_{\mathrm{yx}} & f_{\mathrm{yy}} \end{array}\right]\right)\left[\begin{array}{c} V_{\mathrm{inx}}\left(s\right)\\ V_{\mathrm{iny}}\left(s\right) \end{array}\right]\;G_{\mathrm{HP}}\left(s\right)=\frac{s}{s+\omega_{H}}\;G_{\mathrm{xx}}\left(s\right)≜\frac{x\left(s\right)}{V_{\mathrm{inx}}\left(s\right)}=\frac{\frac{C_{\mathrm{vx}}}{m_{x}}\left(s^{2}+\frac{d_{\mathrm{yy}}}{m_{y}}s+\frac{k_{\mathrm{yy}}}{m_{y}}\right)}{s^{4}+\left(4\Omega_{z}^{2}+\frac{k_{\mathrm{xx}}}{m_{x}}+\frac{k_{\mathrm{yy}}}{m_{y}}\right)s^{2}+2\Omega_{z}\left(\frac{k_{\mathrm{yx}}}{m_{y}}-\frac{k_{\mathrm{xy}}}{m_{x}}\right)s+\frac{k_{\mathrm{xx}}k_{\mathrm{yy}}-k_{\mathrm{xy}}k_{\mathrm{yx}}}{m_{x}m_{y}}}\;G_{\mathrm{xy}}\left(s\right)≜\frac{x\left(s\right)}{V_{\mathrm{iny}}\left(s\right)}=\frac{\frac{C_{\mathrm{vy}}}{m_{y}}\left(2\Omega_{z}s-\frac{k_{\mathrm{xy}}}{m_{x}}\right)}{s^{4}+\left(4\Omega_{z}^{2}+\frac{k_{\mathrm{xx}}}{m_{x}}+\frac{k_{\mathrm{yy}}}{m_{y}}\right)s^{2}+2\Omega_{z}\left(\frac{k_{\mathrm{yx}}}{m_{y}}-\frac{k_{\mathrm{xy}}}{m_{x}}\right)s+\frac{k_{\mathrm{xx}}k_{\mathrm{yy}}-k_{\mathrm{xy}}k_{\mathrm{yx}}}{m_{x}m_{y}}}\;G_{\mathrm{yx}}\left(s\right)≜\frac{y\left(s\right)}{V_{\mathrm{inx}}\left(s\right)}=\frac{\frac{C_{\mathrm{vx}}}{m_{x}}\left(-2\Omega_{z}s-\frac{k_{\mathrm{yx}}}{m_{y}}\right)}{s^{4}+\left(4\Omega_{z}^{2}+\frac{k_{\mathrm{xx}}}{m_{x}}+\frac{k_{\mathrm{yy}}}{m_{y}}\right)s^{2}+2\Omega_{z}\left(\frac{k_{\mathrm{yx}}}{m_{y}}-\frac{k_{\mathrm{xy}}}{m_{x}}\right)s+\frac{k_{\mathrm{xx}}k_{\mathrm{yy}}-k_{\mathrm{xy}}k_{\mathrm{yx}}}{m_{x}m_{y}}}\;G_{\mathrm{yy}}\left(s\right)≜\frac{y\left(s\right)}{V_{\mathrm{iny}}\left(s\right)}=\frac{\frac{C_{\mathrm{vy}}}{m_{y}}\left(s^{2}+\frac{d_{\mathrm{xx}}}{m_{x}}s+\frac{k_{\mathrm{xx}}}{m_{x}}\right)}{s^{4}+\left(4\Omega_{z}^{2}+\frac{k_{\mathrm{xx}}}{m_{x}}+\frac{k_{\mathrm{yy}}}{m_{y}}\right)s^{2}+2\Omega_{z}\left(\frac{k_{\mathrm{yx}}}{m_{y}}-\frac{k_{\mathrm{xy}}}{m_{x}}\right)s+\frac{k_{\mathrm{xx}}k_{\mathrm{yy}}-k_{\mathrm{xy}}k_{\mathrm{yx}}}{m_{x}m_{y}}}$$

The transfer functions $G_{\mathrm{xx}}\left(s\right)$ and $G_{\mathrm{yx}}\left(s\right)$ defined in Equation (2) correspond to the experimental results shown in lines 1 and 2 in Figure 14. $G_{\mathrm{xy}}\left(s\right)$ and $G_{\mathrm{yy}}\left(s\right)$ also correspond to the experimental results shown in lines 1 and 2 in Figure 15. Because the designated proof mass motion is translational, $m_{x}$ and $m_{y}$ are almost the same. Additionally, as shown in Equation (2), the value of the proof mass alone does not affect the frequency response of the system. Therefore, we obtained its value $2.5\times 10^{-5}\;\mathrm{kg}$ from the device dimensions and FEM simulations. Using this mass value, the transfer functions $G_{\mathrm{xx}}\left(s\right)$ – $G_{\mathrm{yy}}\left(s\right)$ defined in Equation (2), and the experimental results shown in Figure 14 and Figure 15, we performed system identification processes to obtain the values for the following parameters: force conversion factors $C_{\mathrm{vx}}\approx C_{\mathrm{vy}}\approx 5\times 10^{-4}\;N/V$; spring constants $k_{\mathrm{xx}}\approx 2.57\times 10^{4}\;N/m$, $k_{\mathrm{xy}}\approx 203.6\;N/m$, $k_{\mathrm{yx}}\approx 160.8\;N/m$, and $k_{\mathrm{yy}}\approx 3.04\times 10^{4}\;N/m$; and damping coefficients $d_{\mathrm{xx}}\approx 0.037\;\mathrm{Ns}/m$ and $d_{\mathrm{yy}}\approx 0.0195\;\mathrm{Ns}/m$. 

**Figure 16:**
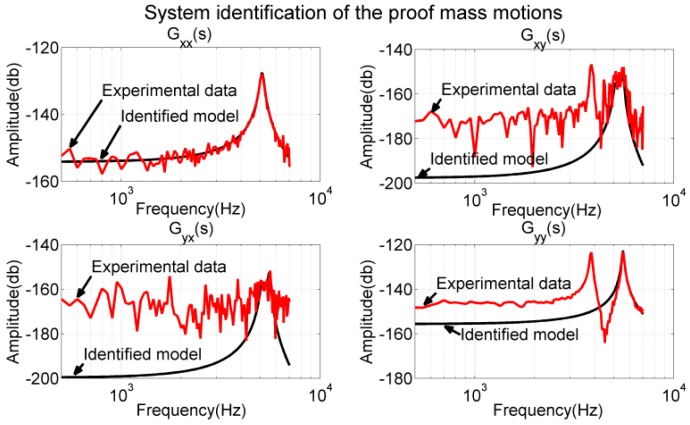
System identification for models $G_{\mathrm{xx}}\left(s\right)-G_{\mathrm{yy}}\left(s\right)$. The identified models matched well with the measurements of the proof mass displacement near the resonant frequencies.

The experimental data and identified dynamic models are presented in Figure 16 for comparison, where the experimental data are represented by the red plot and the frequency response of the identified models is represented by the black plot. As shown in the plot, the proposed models matched well with the experimental data near the designated resonant frequencies. The response of $G_{\mathrm{xy}}\left(s\right)$ and $G_{\mathrm{yx}}\left(s\right)$ did not match well with the experimental data in the low-frequency region because our motion sensing equipment has limited resolution (>5 nm, −170 dB). The response of $G_{\mathrm{yy}}\left(s\right)$ and the experimental data did not match in the low-frequency region because of the interference mode, and this is discussed subsequently.

**Figure 17:**
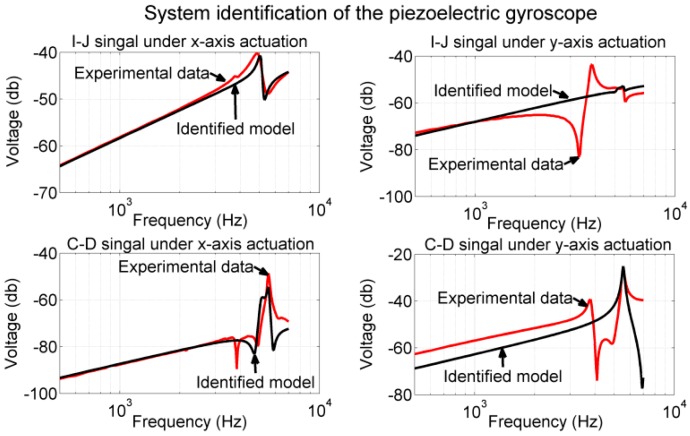
System identification for the overall system. Identified models matched well with the measurements of the sense electrode near the resonant frequencies.

Using the frequency response of the proof mass displacement and voltage outputs shown in Figure 14 and Figure 15, we performed the system identifications to obtain the feedthrough terms and displacement conversion factors. Because all the measured signals exhibited a slope of 20 dB/decade, without losing much accuracy, we assumed the corner frequency $\omega_{H}$ to be $6.28\times 10^{4}\;\mathrm{rad}/s$. With this corner frequency, we identified the remaining system parameters as follows: displacement conversion factors $C_{\mathrm{mx}}\approx 3.33\times 10^{4}\;V/m$ and $C_{\mathrm{my}}\approx 1.56\times 10^{4}\;V/m$; and electrical feedthroughs $f_{\mathrm{xx}}\approx 11.5\;\mathrm{mV}$, $f_{\mathrm{xy}}\approx 4\;\mathrm{mV}$, $f_{\mathrm{yx}}\approx 0.45\;\mathrm{mV}$, and $f_{\mathrm{yy}}\approx 4.7\;\mathrm{mV}$. Using all the identified parameters, we simulated the frequency response of the overall system (the dynamic model shown in Equation (1)) and compared the results with their corresponding experimental data shown in lines 3 and 4 in Figure 14 and Figure 15. As illustrated in Figure 17, the identified model matched well with the experimental data near the resonant frequency. Large deviations were observed at the other frequencies under y-axis actuations, which is discussed in the next section.

### 5.3. Sensitivity of Angular Rate Measurement

**Figure 18:**
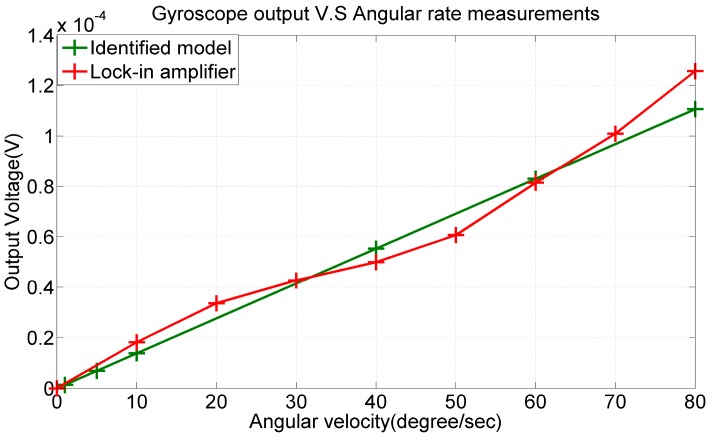
Output voltage versus angular rate. Sensitivity and nonlinearity from the experimental data were 1.82 μV/deg/s and 9.5% FSO, respectively.

In this experiment, electrodes *A* and *B* were given a sinusoidal voltage with a 1-V amplitude and 5.55-kHz frequency but a 180° phase difference. The fabricated PZT gyroscope was placed on a rotation stage with the angular rate ranging from 0°/s to 80°/s. The voltage output of the electrode pair (*I*, *J*) was recorded and processed to null out the quadrature error and electrical feedthrough in order to calculate the angular rate of the stage. In Figure 18, the experimental data are represented by the red plot, and the output signal predicted by the identified gyroscope model is represented by the green plot. 

According to the results, the sensitivity and nonlinearity obtained from the experimental data were 1.82 μV/deg/s and 9.5% FSO, respectively, whereas the sensitivity predicted by the identified model was 1.38 μV/deg/s. Notably, in the aforementioned FEM simulations, the quality factor was 50 [28], the two designated resonant frequencies were 4893 and 4903 Hz, and the predicted sensitivity was 1.38 μV/deg/s in FEM simulations. From the experimental results, the quality factor of the fabricated device was measured at 21.6 in the x-direction and 44.6 in the y-direction. Therefore, the lowered sensitivity can be attributed to the “mode mismatch” [29] and low quality factor.

## 6. Discussion

The resonant frequency of the fabricated device was 5100 Hz in the x-direction and 5550 Hz in the y-direction, whereas the FEM simulations predicted the frequency to be 4908 Hz in the x-direction and 4893 Hz in the y-direction. This mismatched resonant frequencies of the fabricated device led to a 1.82-μV/deg/s device sensitivity which is lower than that of the FEM prediction (1.38 μV/deg/s). When using the dimensions of the fabricated device (Figure 13), the FEM simulations predicted two designated resonant frequencies to be 5192 Hz and 5591 Hz, respectively. Therefore, both the frequency mismatch and lowered sensitivity is mainly came from the device patterning using sandblasting techniques, which engendered various dimension errors and a taper angle on the edge. Notably, both designated mode shapes in the proposed design are in-plane motions, which has an advantage that a different device thickness would not cause problems in designing the mode matching. Therefore, one approach to improve the performance of the current design is to design a gyroscope with less thickness, in which both the device patterning can be accurate and the electrical feedthrough can be minimized. Another approach is to pattern the thick PZT block using low-temperature laser cutting, which may result in a dimension error within 10 μm in this case [30]. 

In the current design, the electrode pair (*A*, *B*) excited both the designated mode shape in the y-direction and the interference mode. The electrode pairs (*E*, *F*) and (*G*, *H*) excited only the designated mode shape in the x-direction. The electrode pair (*C*, *D*) responded to both the designated mode shape in the y-direction and the interference mode. The electrode pair (*I*, *J*) responded to both the designated mode shape in the x-direction and the interference mode. Because the resonant frequency of the interference mode was smaller than those of the two designated modes, all responses of (*C*, *D*), the (*I*, *J*) measurements, and the proof mass displacement in the y-axis were affected by the interference mode in the low-frequency region. The proposed gyroscope model (sixth-order differential equations) could model only the performance of the two designated modes. These results explain the deviation of the identified model $G_{\mathrm{yy}}\left(s\right)$ from the experimental data in the low-frequency region (lower-right plot in Figure 16), and the deviation of the (*I*, *J*), (*C*, *D*) measurements when actuated by electrodes (*A*, *B*) (plots in the right column in Figure 17). 

Because of the existence of the interference mode, the proposed gyroscope design should be operated within the frequency range of 5.5±1 kHz to preserve the linearity of the system response. Ideally, one should design drive electrodes that excite only the designated mode shapes and sense electrodes that respond only to the designated mode shapes. However, this would be a very challenging task, particularly for sense electrodes. One solution to improve the performance of the current design is to implement a symmetrical drive force for actuating the proof mass in the y-axis. In that case, the excitation of the interference mode can be reduced; however, the electrical feedthrough could be worse.

The sensitivity of the fabricated device was calibrated to be 1.82 μV/deg/s without assistance from controls. We may call this “open loop sensitivity”. This value is comparable with values obtained from other PZT gyroscopes [31] but is much lower than the values from conventional quartz gyroscopes; this is because the quartz material has a quality factor of $2\times 10^{6}$, whereas the PZT material is 75 [28]. Although a high quality factor is favorable for sensitivity, it is unfavorable for the control input for tuning the device operating frequency. Conventional gyroscope controls use control inputs to operate the device at its resonant frequency. Therefore, most piezoelectric gyroscopes are made of quartz. Control algorithms involving dual-axis control inputs often employ control inputs to tune the device operating frequency for error compensation. Therefore, from the perspective of system controllability [32], we selected the PZT material, instead of quartz, to create this gyroscope. In summary, although open loop sensitivity is essential for gyroscope performance, it is not the main concern of this design. The trade-off between sensitivity and controllability would be a suitable research topic for the design of gyroscopes. 

The designed resonant frequency of this device was approximately 5 kHz, whereas most piezoelectric gyroscopes operate at a resonant frequency of several tens of kilohertz [4,33]. Notably, a device having the resonant frequency less than 10 kHz is prone to acoustic interference and low quality factors in real applications. The reason that we chose such a low resonant frequency is because algorithms involving dual-axis controls are rather complicated. Besides, implementing a digital controller to verify those algorithms is much easier than implementing an analog controller. A digital controller requires a sampling time at least 10 times faster than the device operating frequency. Considering the hardware capability, we lowered the operating frequency of the device. However, to improve the gyroscope performance, we may increase the device resonant frequency in the long run. 

## 7. Conclusions

In this study, we designed and fabricated a novel PZT gyroscope that can work with advanced control algorithms requiring a dual-axis control input. The proposed design is a cross-type mechanical structure in which the left and right ends of the structure are the proof mass designated for gyroscopic motions. The dimensions and locations of the electrodes were designed carefully such that the drive electrodes can actuate the proof mass efficiently and effectively, sensing electrodes can have the highest sensitivity while responding to the specific shape of flexure deformation, and ground electrodes can minimize the electrical feedthrough. The dimensions of the sensor chip size were determined to be 24.6 mm × 13 mm × 0.2 mm.

According to the experimental results, the resonant frequencies of the fabricated device were 5100 and 5550 Hz in the x- and y-directions, respectively. The sensitivity of the fabricated device was determined to be 1.82 μV/deg/s and the nonlinearity was measured to be 9.5% FSO. The cross-axis mechanical coupling was less than 5.7% in terms of the proof mass displacement. The fabricated device can be operated at 5.5±1 kHz to perform dual-axis actuations and dual-axis position measurements for the gyroscope controls. 

We performed a two-stage system identification to calibrate the performance of the fabricated device. In the identified model, the gyroscopic motions, electromechanical coupling, mechanical coupling, electrostatic coupling, and capacitive output impedance were all included. The proposed model matched well with the experimental data in the frequency range of 5.5±1 kHz.

## Figures and Tables

**Table 1:** FEM simulations of three resonant modes.

Mode Shape	Resonant Frequency (Hz)
Interference mode shape in Y-direction	3428
Designated mode shape in Y-direction	4893
Designated mode shape in X-direction	4908
